# Phenotypic and Genotypic Characterization of *Cutibacterium acnes* Isolated from Shoulder Surgery Reveals Insights into Genetic Diversity

**DOI:** 10.3390/microorganisms11102594

**Published:** 2023-10-20

**Authors:** Mariana Neri Lucas Kurihara, Ingrid Nayara Marcelino Santos, Ana Karolina Antunes Eisen, Giovana Santos Caleiro, Jansen de Araújo, Romário Oliveira de Sales, Antônio Carlos Pignatari, Mauro José Salles

**Affiliations:** 1 Laboratório Especial de Microbiologia Clínica (LEMC), Departamento de Medicina, Escola Paulista de Medicina (EPM), Universidade Federal de Sao Paulo (UNIFESP), Sao Paulo 04025-010, Brazil; mariana.kurihara@hotmail.com (M.N.L.K.); inayarams@gmail.com (I.N.M.S.); accpignatari@gmail.com (A.C.P.); 2 Emerging Viruses Research Laboratory, Institute of Biomedical Sciences, University of Sao Paulo, Sao Paulo 05508-000, Brazil; anaeisen@usp.br (A.K.A.E.); giovanacaleiro@gmail.com (G.S.C.); jansentequila@usp.br (J.d.A.); 3 Albert Einstein Research and Education Institute, Hospital Israelita Albert Einstein, Sao Paulo 05652-900, Brazil; romariosallespva1@gmail.com

**Keywords:** *Cutibacterium acnes*, shoulder surgery, whole genome sequencing, phylotyping, virulence markers, colonization

## Abstract

Specific virulence factors that likely influence *C. acnes* invasion into deep tissues remain to be elucidated. Herein, we describe the frequency of *C. acnes* identification in deep tissue specimens of patients undergoing clean shoulder surgery and assess its phenotypic and genetic traits associated with virulence and antibiotic resistance patterns, compared with isolates from the skin of healthy volunteers. Multiple deep tissue specimens from the bone fragments, tendons, and bursa of 84 otherwise healthy patients undergoing primary clean-open and arthroscopic shoulder surgeries were aseptically collected. The overall yield of tissue sample cultures was 21.5% (55/255), with 11.8% (30/255) identified as *C. acnes* in 27.3% (23/84) of patients. Antibiotic resistance rates were low, with most strains expressing susceptibility to first-line antibiotics, while a few were resistant to penicillin and rifampicin. Phylotypes IB (73.3%) and II (23.3%) were predominant in deep tissue samples. Genomic analysis demonstrated differences in the pangenome of the isolates from the same clade. Even though strains displayed a range of pathogenic markers, such as biofilm formation, patients did not evolve to infection during the 1-year follow-up. This suggests that the presence of polyclonal *C. acnes* in multiple deep tissue samples does not necessarily indicate infection.

## 1. Introduction

*C. acnes* is an aerotolerant, anaerobic, Gram-positive microorganism that plays a key role in the balance of the skin microbiome, particularly in the pilosebaceous unit in the upper regions of the human body, such as the shoulder region [1]. *C. acnes* has been increasingly recognized as a true pathogen following shoulder surgical procedures when associated with implant insertion [2,3]. According to previous studies assessing prosthetic shoulder infections, *C. acnes* has been identified as the leading cause in tissue samples [3,4]. The clinical aspects of orthopedic implant-associated infections (OIAI) caused by *C. acnes* vary widely, mainly from asymptomatic to the presence of local and systemic signs and symptoms of acute inflammation, whereas nonspecific chronic inflammatory symptoms such as local pain and radiological signs of implant loosening have also been commonly reported [2,3,4]. Levy et al., reported that deep tissue specimens yielded *C. acnes* in 20% of asymptomatic patients after shoulder arthroscopy. Therefore, whether *C. acnes* growing from deep tissue specimens of asymptomatic patients undergoing shoulder surgery should be considered a true infection due to the possibility of implant loosening remains a matter of debate.

Despite the progress of recent studies assessing genetic traits, it is still unclear how commensal *C. acnes* contributes to the pathogenesis of OIAI [5,6]. Studies on *C. acnes* genes have identified putative virulence factors associated with extracellular hemolysin and cytotoxin, inflammatory response, biofilm formation, and resistance to antibiotics [7,8,9,10,11,12]. Previous studies have attempted to postulate the invasiveness of *C. acnes* with genetic diversity (subtypes) using multilocus sequence typing (MLST) (types IA_1_, IA_2_, IB, IC, II, and III) and single-locus sequence typing (SLST) [13,14]. Through MLST, types IB and II are commonly found in soft tissue and OIAI infections [15]. Some phylotypes express the lyase hyaluronate lyase (*hyl*), which induces an inflammatory response, whereas others exhibit a higher expression of proteins linked to cell invasion and adhesion at different stages of biofilm formation [8,16]. In addition, the SLST method categorizes isolates into ten major clades, A to E, corresponding to phylotype IA_1_ strains, whereas clades F, G, H, K, and L correspond to phylotypes IA_2_, IC, IB, II, and III, respectively [14,17]. More recently, high-throughput sequencing techniques have uncovered patterns in the genomic traits of *C. acnes,* enabling the recognition of emerging pathogenic features. This allowed the identification of pathogenic and resistance-related genes as well as the prediction of clustered regularly interspaced short palindromic repeats (CRISPRs) and CRISPR-associated genes (*cas*). This includes the use of type I–E CRISPR-Cas systems found in type II *C. acnes*, which serve as an adaptive immune mechanism. This observation suggests genetic stability and resilience against phage-related factors within this phylotype [18,19]. Moreover, pangenome analysis serves as a tool for comparative genomics of the strains within the study or in comparison to a database, providing a more comprehensive insight into accessory genes and genomic conservation [20,21]. While *C. acnes* has been associated with OIAI, a positive tissue culture alone is likely insufficient to trigger an antibiotic treatment [15]. In this context, we sought to investigate the frequency of *C. acnes* yielded from deep tissue specimens in non-infected adults undergoing shoulder surgeries. Additionally, we assessed outcomes over a 12-month follow-up period and conducted a comparative analysis of the specific phenotypic and genotypic traits of *C. acnes* strains, utilizing whole-genome sequencing (WGS), with strains identified on the skin of healthy volunteers.

## 2. Materials and Methods

### 2.1. Study Design and Patients

This experimental and clinical study was conducted on *C. acnes* isolates identified in deep tissues collected from patients undergoing arthroscopic or non-arthroplasty open primary clean shoulder surgeries from July 2019 to January 2020 at Brazilian tertiary hospitals and from skin swabs of healthy volunteers. Three intraoperative deep tissue biopsy specimens were obtained per patient and submitted for microbiological workup. Patients under 18 years old with a previous diagnosis of bone or joint infection, who underwent previous surgeries on the same shoulder, and/or those who had recently used antimicrobials within a period of up to 15 days prior to sample collection were excluded from this study. Healthy volunteers not associated with the hospital were included as controls. Samples were collected from the surface near the pilosebaceous glands in the clavicle region using an eSwab (Copan Italia, Brescia, Italy). This study was approved by the Ethics and Research Committee of the Institution under number 22270619.9.0000.5505, and all participants provided written informed consent. All information regarding the isolates was anonymized. The criteria for the definition of infection caused by *C. acnes* were as follows: contamination or infection according to the number of positive samples and the presence of clinical signs and symptoms of shoulder infection [22,23].

### 2.2. Sample Collection, Laboratory Processing, and Bacterial Identification

Three deep tissue samples from 84 patients were collected from the bone fragments (humeral head, acromion, or distal third of the clavicle), tendon (supraspinatus, long head of the biceps tendon, or conjoint tendon), and bursa (approximately 0.5 cm^3^). Each specimen was collected using separate surgical instruments (sterile scalpels and tweezers) according to the Oxford technique for specimen collection and promptly placed randomly into two thioglycolate medium flasks (Oxoid Ltd., Thermo Fisher Scientific, Boston, MA, USA) and one in a sterile 0.85% saline solution flask [24]. The samples were brought to the microbiology laboratory in a timely manner for no longer than two hours.

**Figure 1:**
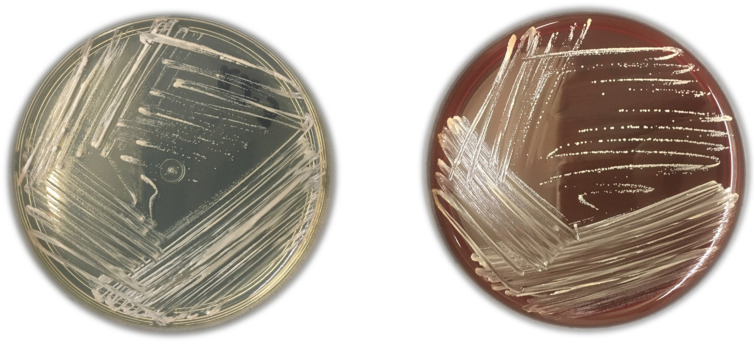
Nonhemolytic *C. acnes* colonies were isolated on Schaedler Agar and Sheep Blood Agar plates.

Sterile deep-tissue samples were incubated in thioglycolate (Oxoid Ltd., Thermo Fisher Scientific, Boston, MA, USA) using anaerobic jars (Oxoid Ltd., Thermo Fisher Scientific, Boston, MA, USA) and visually inspected for microbial growth daily for 14 days. Additionally, sterile 0.85% saline solution tissue samples were placed into tryptic soy broth (TSB) (KASVI^®^, Sao Jose dos Pinhais, Brazil), incubated at 37 °C under aerobic conditions, and visually inspected for 14 days for any growth. TSB and thioglycolate were seeded on sheep blood agar plates (BioMérieux, Marcy l’Étoile, France) and Schaedler Agar (Merck, Darmstadt, Germany) at 37 °C under anaerobic conditions for 14 days. Hemolysis was recorded if zones of clearance were observed on sheep blood agar plates [25,26]. Finally, the colonies shown in Figure 1 were stored in TSB (KASVI^®^, Brazil) with 30% glycerol at −80 °C (Figure 1). For the control group, all skin swabs were vortexed for 10 s, seeded onto sheep’s blood agar plates (BioMérieux, Marcy l’Étoile, France) and Schaedler Agar (Merck, Darmstadt, Germany), and incubated at 37 °C under anaerobic conditions for 14 days.

Microbial identification was carried out by the matrix-assisted laser ionization-desorption-time-of-flight (MALDI-TOF MS) technique using a Microflex LT spectrometer and the BiotyperTM 3.3 software package (Bruker Daltonics^TM^, Billerica, MA, USA). Furthermore, isolates of *C. acnes*, *Cutibacterium granulosum*, *Cutibacterium avidum*, *Corynebacterium simulans*, and *Propionibacterium acidifaciens* had their DNA extracted using Chelex 100^®^ resin (BioRad, Hercules, CA, USA), and the identification was confirmed through amplification reactions for the *PArA-*1 gene of the 16S rRNA region of *C. acnes*. *C. granulosum*, *C. avidum*, *C. simulans*, *P. acidifaciens*, and deionized H_2_O were used as negative controls in the reaction. In separate tubes, Top Taq^®^ Master Mix Kit (Qiagen, Hilden, Germany), sterile water, and the primer set described by Emma Barnard et al. (2015) targeting the 16S rRNA region were prepared with 1 μL of genomic DNA to a final volume of 20 μL, following the denaturation protocol previously described by Emma Barnard et al. (2015) [27]. PCR was performed using a Mastercycler Gradient Thermocycler (Eppendorf, Hamburg, Germany). PCR products were analyzed by electrophoresis on 1.5% agarose gels stained with UniSafe Dye^®^ (Uniscience, Sao Paulo, Brazil) and were considered positive when the fragment amplification matched the primer base pairs described [27].

### 2.3. Multiplex-Touchdown Polymerase Chain Reaction (Multiplex Touchdown PCR) Phylotyping

*C. acnes* phylotyping was performed by testing the DNA extracted from the previous PCR reaction. DNA was subjected to a new amplification reaction using *PAMp* primers specific to *C. acnes*. Additionally, the reaction included the set of *PAMp* primers and the denaturation sequence, as described by Barnard et al. in 2015. PCR was performed in a Mastercycler Gradient Thermocycler (Eppendorf, Hamburg, Germany). PCR products were analyzed by electrophoresis on 2% agarose gels stained with UniSafe Dye^®^ (Uniscience, Brazil) and were considered positive when the fragment amplification matched the primer base pairs previously described [27].

### 2.4. Antibiotic Susceptibility Testing and Detection of Resistance Factors

Suspension in sterile 0.85% saline solution at a density of 1.0 on the McFarland scale was used to determine the susceptibility pattern of each *C. acnes* isolate. The inoculum was immediately seeded using a sterile swab onto an Anaerinsol-S (Probac do Brasil, Sao Paulo, Brazil) culture medium plate. The Etest strips (BioMérieux, Marcy-l’Étoile, France) were tested for metronidazole, vancomycin, penicillin, rifampicin, linezolid, daptomycin, clindamycin, ceftriaxone, ciprofloxacin, and amoxicillin/clavulanic acid. All the plates were incubated at 37 °C in 4–10% CO_2_ for 72 h under anaerobic conditions. Plates were examined for the minimum inhibitory concentration (MIC) on the Etest^®^ strips following the recommendations of the Brazilian Committee on Antimicrobial Susceptibility Testing (B_R_CAST, 2023) and compared to the Clinical Laboratory Standards Institute (CLSI, 2023) recommendations [28,29].

### 2.5. Whole Genome Sequencing and Bioinformatic Tools

Eleven *C. acnes* isolates were chosen for WGS to further explore the relevant genomic features related to specific clinical cases. Bacterial DNA was purified using the ZymoBIOMICS DNA Miniprep Kit (Zymo Research, Orange, CA, USA), and the concentration was assessed using a Qubit^®^ 2.0 fluorometer (Invitrogen, Carlsbad, CA, USA). The Ion Sequencing Kit v2.0 was used for all sequencing reactions, following the manufacturer’s recommendations. The sequence reads generated were then compared and annotated using the Bacterial and Viral Bioinformatics Resource Center (BV-BRC) database https://www.bv-brc.org/ (accessed on 10 February 2023), as well as the phylogenetic tree. This tree was established by comparing the sequences to their respective reference strains, with strain IA_2_ being compared to the NCBI:txid1114969 entry, designated as the P. acn31 strain. The assembly of the strains is publicly available from the National Centre for Biotechnology Information (NCBI) under Genomes accession numbers JASPKO000000000, JASPKN000000000, JASPKM000000000, JASPKL000000000, JASPKK000000000, JASPKJ000000000, JASPKI000000000, JASPKH000000000, JASPKG000000000, JASPKF000000000, and JASPKE000000000. Genes mediating antimicrobial resistance were analyzed using CGE ResFinder 4.1 https://cge.food.dtu.dk/services/ResFinder/ (accessed on 10 February 2023). Single-locus sequence typing (SLST) assignment was performed http://medbac.dk/slst_server_script.html (accessed on 10 February 2023). MLST of the samples was performed on PubMLST https://pubmlst.org/organisms/cutibacterium-acnes (accessed on 11 February 2023) and MLST 2.0 https://cge.food.dtu.dk/services/MLST/ (accessed on 11 February 2023). CrisprCas was determined using CRISPRCasFinder https://crisprcas.i2bc.paris-saclay.fr/CrisprCasFinder/Index (accessed on 9 March 2023). All genomes were reannotated with Prokka v1.13.3 using standard options [30]. We used Roary v3.12.0 to perform a core-pan genome analysis of the 11 *C. acnes* strains [31]. The core genome is defined as at least 95% of the genome of the strain. Genomic comparison was conducted between each strain and its corresponding reference ATCC strain using the CGView Comparison Tool [32]. The sequence features, gene and protein names, COG (Cluster of Orthologous Groups) category assignments, and sequence composition characteristics adhered to the script provided the webpage https://paulstothard.github.io/cgview_comparison_tool/index.html (accessed on 8 August 2023). [30].

### 2.6. Statistical Analysis

For statistical analysis, an association with qualitative characteristics was performed using the chi-square test and Fisher’s exact test, confirmed by Pearson, and tested for two samples. The difference was considered statistically significant if the value was less than or equal to 0.05. All data were analyzed using SPSS version 23 (IBM-SPSS Inc., Chicago, IL, USA).

## 3. Results

### 3.1. Study Population, Microbiological Results, and Confirmation of C. acnes Strains

Overall, 84 patients who underwent primary clean shoulder surgery with sterile deep tissue specimens were included in this study. Among them, 54% (45/84) were male, with a mean age of 51 years (SD ± 17 years). Relevant comorbidities included cardiovascular disease (42%), diabetes mellitus (30%), and smoking habits (13%). Arthrotomies and arthroscopic surgeries were performed on 35.7% (30/84) and 64.3% (54/84) of patients, respectively. Male sex was a unique variable associated with a greater likelihood of *C. acnes* growth (*p* = 0.005). The demographic and clinical characteristics of this study population are summarized in Table 1.

The identification match using MALDI-TOF MS and molecular analysis by *PArA*-1 confirmed the presence of *C. acnes* in shoulder tissue specimens and skin swabs of healthy volunteers. The presence of the *PArA*-1 gene was absent in the isolates of *C. granulosum*, *C. simulans*, and *P. acidifaciens*, as expected.

A total of 255 tissue specimens were collected, yielding a positive bacterial culture rate of 21.5%. Among the positive specimens, 30 (11.8%) were identified as *C. acnes* in 23 patients (27.3%). Of these, *C. acnes* was recovered from two tissue specimens from five patients (6, 8, 17, 34, 42) and from three specimens from patient 29. *C. acnes* was identified in four healthy volunteers who underwent superficial clavicular region swabs. Prior researchers have examined hemolysis as a potential marker for pathogenicity; however, it is important to note that zones of clearance were not detected in our isolates.

### 3.2. Antimicrobial Susceptibility Test of C. acnes Isolates

Overall, 27 *C. acnes* strains were tested, of which 100% showed the predicted resistance to metronidazole (MIC > 256 mg/L). The strains were mostly susceptible to first-line antibiotics, showing lower levels of MIC < 0.016 mg/L for clindamycin and amoxicillin in 52% (*n* = 14/27) and 89% (*n* = 24/27) of cases, respectively. Similar results were observed for penicillin, except for strain 42.2, which demonstrated resistance (MIC = 0.64). All the strains tested showed susceptibility to vancomycin. Resistance to rifampicin was identified in one strain with an MIC > 256 mg/L (strain 22.3). Ciprofloxacin MIC values ranged from 0.25 to >32 (strain 30.3). It was not possible to assess the susceptibility of the three strains (6.3, 76.1, and 83.3). Susceptibility profile distributions were compared using the B_R_CAST and CLSI breakpoint tables (Supplementary Table S1).

### 3.3. Phylotyping of C. acnes Strains by Molecular Typing and Whole-Genome Sequencing

A total of 30 strains of *C. acnes* isolated from shoulder specimens and four from healthy skin swabs were identified by multiplex PCR; phylotypes type IA_1_ (*n* = 1), IB (*n* = 21), II (*n* = 8), and IA_1_ (*n* = 1), IA_2_ (*n* = 2), and IB (*n* = 1). Of the 34 strains, 11 were chosen for next-generation sequencing (NGS) and were compared with the reference genome sequences of *C. acnes* strains available in GenBank. The genomes of nine strains from the tissue specimens belonged to three different phylotypes: mainly type IB (*n* = 7), phylotype IA_2_ (*n* = 1), and type II (*n* = 1). Genome sequencing of the skin strains CACO1 and CACO15 belonged to phylotypes IA_1_ and IB, respectively (Table 2).

Overall, the SLST analysis revealed the predominance of type H1 (IB) in shoulder tissue specimens (7/9) and healthy skin. Additional SLST phylotypes were designated as K1 (II) and F1 (IA_2_), found in shoulder tissue specimens from the same patient, and A1 (IA_1_) from a skin swab (Table 2).

### 3.4. Whole Genome Sequencing of C. acnes Strains

**Figure 2:**
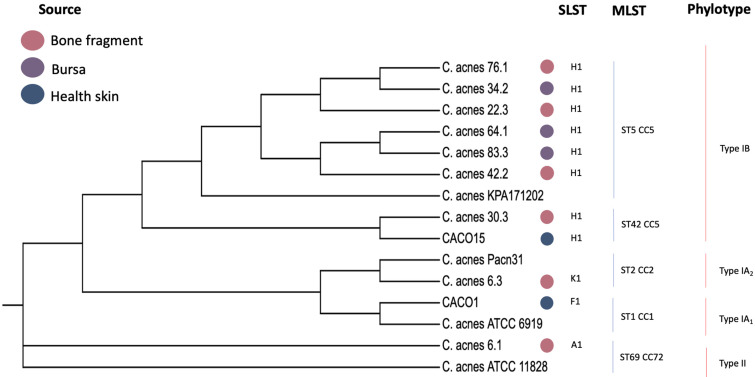
The phylogenetic tree of *C. acnes* was sequenced with the source, SLST, MLST, and phylotype distribution.

A comparison of the phylogenomic analysis of the 11 strains studied (nine from shoulder tissues and two from skin swabs) with the genome sequences of *C. acnes* strains available in GenBank revealed that no clear separation was identified regarding the source of bacteria (healthy skin from shoulder deep tissue specimens) (Figure 2). Plasmids potentially harboring resistance genes were searched but could not be detected. The most common type of clonal complex (CC) identified in tissue specimens was CC5 (77.8%), followed by CC2 and CC72. The CCs of the skin isolates were CC5 and CC1.

Notably, patient 6 had one isolate (6.1) with a genome length of 2,470,359 bp that belonged to ST69 CC72 type II (SLST K1). The presence of the CAS-TypeIE CRISPR-Cas system (Start 810,485–End 818,700) was observed, which was expected for *C. acnes* type II [20]. Interestingly, another strain (6.3) was isolated from the same patient but from a different tissue source, with a genome length of 2,467,134 bp belonging to the ST2 CC2 type IA_2_ (SLST F1). *C. acnes* isolates from different patients (22.3, 34.2, 42.2, 64.1, 76.1, and 83.3) were associated with ST5 CC5 type IB (SLST type H1). Two patients presented one isolate (30.3 and CACO15) associated with ST42 CC5 type IB (SLST type H1), but with a variation in genome length, G+C content, and protein-coding sequences (CDS). We observed an isolate (CACO1) with a genome length of 2,465,755 bp, associated with ST1 CC1 type IA_1_ (SLST type F1). Genome length, G+C content, and protein-coding sequences (CDS).

Putative virulence factor genes for hemolysin and inflammation, such as *tly* and *hyl*, were not identified in any of our strains. On the other hand, genes involved in biofilm formation, including *Lpq*B; genes associated with cellular growth and cell wall homeostasis; heat shock proteins such as *Dna*K, *Dna*J, and *Gro*EL chaperonin; and the elongation factors EF-Tu, EF-G, and CAMP factor, have been identified [33,34]. The *Flp* pili gene (adherence and persistence) was present in all samples studied, even if the strains were plasmid-negative [35].

**Figure 3:**
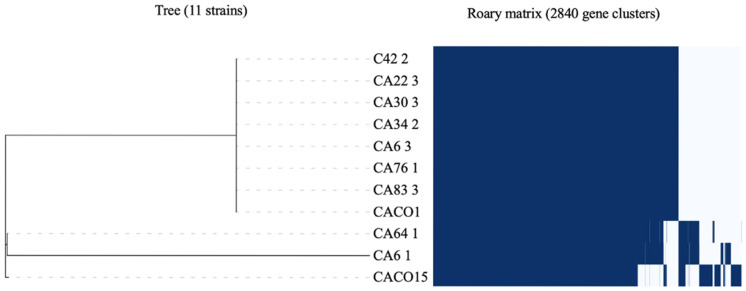
Genetic relatedness of 11 *C. acnes* strains from deep tissue shoulder surgeries. Phylogenetic tree and Roary pan-genome matrix showing the presence (dark blue color) and absence (white color) of accessory genes in the genomes, with 2840 gene clusters.

To comprehensively analyze the genomic conservation across the 11 strains, the total coding sequences were utilized to determine both the pan and core genomes. Overall, 2840 genes were identified within the pangenome, with 1886 genes present in the core genome. Moreover, the presence of 954 accessory genes likely contributed to the genetic variability within the samples studied. Interestingly, strains 64.1, 6.1, and CACO15 exhibited variability in their gene content, as illustrated in Figure 3. This variation in the accessory genes accounts for the observed disparities in the genome of each phylotype and can be further explored in the Supplementary Material (Table S2).

In addition, four circular genomic comparison maps were generated, each presenting a high degree of similarity (>60%) between the studied samples and the reference strain matching its phylotype, as shown in the supplemental material (Figure S1A–D). In each circular genome, we observed that the outermost rings in the map showed the COG functional categories for the reverse strand, reverse strand feature, forward strand feature, and COG functional categories for the forward strand. The majority of COGs are related to bacterial metabolism, such as coenzyme transport, lipid transport, and metabolism, as well as the biosynthesis, transport, and catabolism of secondary metabolites, as depicted in the supplemental material (Figure S1A–D) [20].

## 4. Discussion

Considering that *C. acnes* is a part of the skin microbiota, we aimed to contribute to the understanding of the microbiological features of *C. acnes* isolated from tissue specimens of patients undergoing clean shoulder surgery. We assessed polyclonal phenotypic and genetic traits, including antibiotic susceptibility patterns, phylotyping, virulence, resistance genes, and pan- and core-genome context. Despite collecting deep tissue specimens near pilosebaceous glands, *C. acnes* was isolated from 11.8% of tissue biopsies in 27.3% of asymptomatic patients. The rates of *C. acnes* recovery from patients undergoing arthrotomy and arthroscopy surgery in previous studies were similar, ranging from 20% to 36% [36,37].

Prolonged anaerobic incubation presents challenges related to contamination or low yields of *C. acnes* [24]. The identification of *C. acnes* was a critical aspect of this study. Currently, MALDI TOF-MS facilitates bacterial identification and is cost-effective [38]. However, molecular confirmation using the 16S rRNA region is a valuable tool to support the identification of the studied samples, as it is specific to bacteria and has been used to identify *C. acnes* in skin [39]. In contrast to previous studies, phylotyping analysis by PCR did not meet our expectations when compared to WGS, due to the non-specific fragment alignment [27]. Here, we report the concordance between NGS and MALDI TOF MS for identification.

This study presents the prevalence of phylotypes IB (73.3%) and II (23.3%), clades H1 and CC5. These genetic profiles have exhibited an overrepresentation in cases of OIAI and shoulder SSI but also in healthy skin [7,16,40]. Nevertheless, our study identified that phylotype IB ST5 CC5 is recurrently found on healthy skin and demonstrates a significant correlation with OIAI, notably in the context of shoulder surgeries [41]. Phylotypes CC36 (type IB) and CC53 (type II) have been associated with prosthetic joint infections [7,18]. Notably, the type II strain studied was part of the CC72 strain, which has been described in both healthy skin and clinical infection cases; however, its precise significance remains debatable [42]. The pathogenic potential of phylotypes IB and II involves microbial infiltration through the open superficial skin incision and their crucial role in adhering to orthopedic implants, contributing to reinfection or persistent infection [43]. Additionally, the type II strain identified exhibits the CRISPR/Cas system, an adaptive differentiation among this phylotype that confers immunity against acquired mobile elements and could be a marker for strain differentiation [20,44].

We report the predominance of SLST type H1 in shoulder deep tissues of asymptomatic patients, while Salar Vidal et al. compared the genomic traits of *C. acnes* strains isolated from prosthetic joint infections (PJI) cases according to clinical outcomes from several European hospitals; type H1 was associated with relapse cases, particularly in hip PJI cases [39,45]. Type H1 was previously described in patients of medical emergency centers, predominantly in the hip, whereas isolates belonging to clade K have been described in plastic surgery settings, specifically in prostatic tissue [6,35,41,46,47]. Clade A1 (IA_1_) is commonly associated with severe acne skin conditions, as are clades C (IA_1_) and F (IA_2_) [6,46,48,49]. Although we observed the predominance of a single phylotype in each patient analyzed, different *C. acnes* phylotypes were found within the deep tissue specimens of three patients: six (IA_2_ and II, clade K1 and F1), eight, and 29 (II and IB). Bumgarner et al. observed polyclonal phylotype diversity of *C. acnes* in a deep tissue sample of 45% of patients undergoing revision shoulder arthroplasty to adapt to the microenvironment of the skin [44]. Indeed, if different *C. acnes* phylotypes are found, this may reflect polyclonal infection or contamination likely occurring during surgery or sample transportation [16,48].

Genomic analysis of the 11 strains revealed high similarity to the reference genomes. *C. acnes* evaluated in this study showed the presence of fimbrial low-molecular-weight protein (*flp*) pili, which are predicted to be involved in adhesion and colonization and have been regarded as a possible marker for virulence in shoulder PJI cases, especially among phylotypes IA and II [35]. We also observed the presence of the lipoprotein *Lpq*B gene, which functions as a modulator of the MtrAB two-component system via signal transduction, is involved in cellular growth and cell wall homeostasis, and affects biofilm formation. Moreover, the GroEL chaperonin and elongation factors EF-Tu, EF-G, and CAMP factor, which are associated with biofilm production, were also predicted [33,34]. The *deoR* gene, implicated in pathogenesis, was detected in all of our strains, albeit with a higher prevalence in type IA_2_ and II strains [16]. Although genes *tly* (putative hemolysin), *hyl* (hyaluronate lyase), and *gehA* (lipase) are known virulence factors, they were not detected in our samples, aligning with the non-hemolytic profile expressed by our isolates, which supports the possibility of non-pathogenic strains [11,16,20,49]. *C. acnes*-presenting hemolysis was previously related to an increased pathogenic potential and clinical outcome [25,26]. However, a previous study showed that the expression of the aβ-hemolytic profile does not correlate with phylotypes or clinically significant infections [50].

The analysis of both the pan and core genomes of our samples differed from that of a previously published study that investigated 255 *C. acnes* isolates. This prior study reported 6240 genes in the pan-genome and 1194 genes in the core genome with a threshold of 100%, whose genomes maintain their similarity [20]. This extensive analysis of isolates contributed to the exploration of genetic diversity within the species’ pan-genome while concurrently observing a reduction in core genome genes [20]. While a direct comparison with our study is limited by differences in algorithms, it is evident that our strains are generally well conserved in the pan genome, with only three displaying variations that could be related to acquired gene events, as discussed by Rocha et al., 2022 [51]. Analysis of the accessory genes revealed that most of them were associated with general bacterial metabolism. Of particular importance is the presence of the Response Regulator *MprA*, which is responsible for maintaining cellular equilibrium under stress and the persistence of infection in all isolates. Interestingly, we observed the absence of the S-ribosylhomocysteine lyase gene (*luxS_2*), which is crucial for quorum sensing, in isolates 6.1, 64.1, and CACO15. However, these isolates were unique in presenting the Antiseptic Resistance Protein, represented by the *qacA* gene. Moreover, the Transcriptional Regulatory Protein *LiaR*, which is linked to cellular stress regulation, was exclusively identified in isolate 6.1. Notably, even though strains 64.1 and CACO15 belong to phylotype IB, they possess distinct sequence types (STs), specifically ST5 CC5 and ST42 CC5. Thus, the presence of genes pivotal for bacterial survival and pathogenicity has been described in bacteria from different clades. This phenomenon has also been previously observed in other bacterial species as well [16,52,53,54].

The antimicrobial susceptibility patterns of the analyzed *C. acnes* strains revealed low rates of resistance, suggesting that susceptibility to antimicrobials remained intact [13,15]. While previous studies have reported resistance mostly to clindamycin in shoulder surgery patients, only samples 22.3 and 30.3 expressed resistance to rifampicin and ciprofloxacin, respectively. Rifampicin has been described as an important antimicrobial agent for biofilm eradication, and its resistance has been reported in *C. acnes* and *Cutibacterium namnentese* isolates that harbor the *rpoB* gene [55,56]. We identified different point mutations (A228E and L470S) in the *rpoB* gene of strain 22.3, which are yet to be further studied.

The present study has several limitations. It is possible that the bacteria identified in our study originated from skin contamination during surgery, sample collection, transportation, or laboratory processing, despite our efforts to minimize contamination during sample collection and ensure prompt delivery to the laboratory. These findings highlight the inherent genetic variability of *C. acnes* within the natural microbiota. The overall cohort size, comprising both patients and samples, was relatively modest, which may have resulted in an underestimation of the recovery rates of this bacterium in the clean deep tissue samples. Moreover, the follow-up period for patient assessment was extended to only one year. While there is existing research suggesting the adequacy of this timeframe for certain assessments, it may have limited our capacity to thoroughly examine the results over an extended period [57]. Lastly, it should be noted that not all strains underwent comprehensive WGS analysis, which could potentially have unveiled pivotal genomic traits.

Our findings indicate that *C. acnes* cultures exhibit a polyclonal nature and lack evidence of pathogenic or resistance gene expression. While MLST and SLST analyses have revealed the presence of specific significant genetic markers, from both phenotypic and clinical standpoints, our isolates do not appear to be associated with infections, mirroring strains identified in the epidermis of healthy individuals. Therefore, further investigation with the sequencing of a larger sample size and additional diagnostic tests is necessary to confirm our findings and fully understand the relationship between *C. acnes* and healthy skin and surgeries.

## 5. Conclusions

Our study offers valuable insights into the phenotypic and genetic characteristics of *C. acnes* strains isolated from deep tissues in the context of shoulder surgeries. Molecular analysis reveals that *C. acnes* strains from non-infected shoulder surgeries demonstrate potential invasiveness, as evidenced by the presence of virulence factors related to adhesion and biofilm formation genes. In contrast, the typical pathogenic markers associated with postoperative infections, such as extracellular hemolysin, cytotoxin, and antibiotic resistance, were frequently absent in our isolates. Furthermore, our findings highlight the diversity of *C. acnes* strains, with different phylotypes present in multiple specimens. These strains showed no association with infections, as indicated by their close genetic resemblance to *C. acnes* strains found on healthy skin. This underscores the need for a more comprehensive understanding of the role of *C. acnes* in surgical contexts and emphasizes the potential significance of factors beyond bacterial presence in determining infection outcomes.

## Figures and Tables

**Table 1:** Demographics and clinical characteristics of the 84 patients included in this study.

Demographics and Clinical Characteristics	Patients			*p*-Value
	*C. acnes* Positive on Cultures % (*n* = 23)	*C. acnes* Negative on Cultures % (*n* = 61)	Total % (*n* = 84)	
Gender				
Male	78 (18)	44 (27)	54 (45)	0.005
Female	22 (5)	56 (34)	46 (39)	-
Mean age (SD ^#^ ±)	44.6 (±16)	54.1 (±15)	51.5 (±17)	0.005
Arthrotomy (*n* = 30)	50.5 (±15.2)	59.8 (±18.9)	57.3 (±15)	
Arthroscopy *(n* = 54)	40.8 (±14.7)	50.9 (±14.4)	48.3 (±19)	
Comorbidities and habits				
Smoking habits	13 (3)	13.1 (8)	13 (11)	0.723
Chronic alcoholism	13 (3)	14.7 (9)	14.2 (12)	0.880
Cardiovascular disease	34.7 (8)	44.2 (27)	41.6 (35)	0.590
Diabetes mellitus	13 (3)	36 (22)	29.7 (25)	0.073
Types of surgery ^a^				
Arthroscopy	47.8 (11)	37.7 (23)	40.5 (34)	0.550
Arthrotomy	52.2 (12)	62.2 (38)	59.5 (50)	-
Type of orthopedic implant ^b^				
Metallic	56.5 (13)	47.5 (29)	50 (42)	0.833
Non-metallic	43.5 (10)	45.9 (28)	45.2 (38)	-
Signs of surgical site infection	4.4 (1)	3.2 (2)	3.5 (3)	0.671
Intraoperative abnormality	0 (0)	0 (0)	0 (0)	-
Clinical follow-up: Infection ^c^				
No evidence of infection	95.6 (22)	93.4 (57)	94 (79)	0.723
Probable contamination	4.4 (1)	3.2 (2)	3.5 (3)	0.671
Probable infection	0 (0)	1.6 (1)	1.1 (1)	-
Confirmed infection	0 (0)	1.6 (1)	1.1 (1) ^$^	-

^a^ *C. acnes*: Cutibacterium acnes; ^#^ SD: standard deviation; ^b^ Four samples not described; ^c^ Six samples not described. ^$^ Patient with clinical signs and symptoms of acute superficial surgical site infection, but tissue cultures are negative.

**Table 2:** Sample source, genotypic results depicting the SLST ^#^ and MLST ^&^ (WGS) subtypes, and relevant genes in the genome of eleven *C. acnes* isolates.

Isolate ID	Sample Source	^#^ SLST	^&^ MLST	Virulence Factor		Biofilm Formation							CRISPR-Cas Systems			Stress Response			Resistance
				*clpS*	*dppB*	*hyl*	* PNAG *	* EF-Tu *	*EF-G*	*rcsB*	*luxS2*	*acsA*	*luxS2*	*casC*	*fucA*	*MprA*	*LiaR*	*mprA1*	*qacA*
6.1	Bone fragment	K1	ST69 CC72 type II	1	1	0	0	1	1	1	0	1	0	1	0	1	1	0	0
6.3	Bone fragment	F1	ST2 CC2 type IA_2_	1	0	0	0	1	1	0	1	0	1	0	1	0	0	1	1
22.3	Bone fragment	H1	ST5 CC5 type IB	0	0	0	0	1	1	0	1	0	1	0	1	0	0	1	1
30.3	Bone fragment	H1	ST42 CC5 type IB	0	0	0	0	1	1	0	1	0	1	0	1	0	0	1	1
34.2	Bursa	H1	ST5 CC5 type IB	1	0	0	0	1	1	0	1	0	1	0	1	0	0	1	1
42.2	Bone fragment	H1	ST5 CC5 type IB	0	0	0	0	1	1	0	1	0	1	0	1	0	0	1	1
64.1	Bursa	H1	ST5 CC5 type IB	0	0	0	0	1	1	0	0	0	0	0	0	1	0	0	0
76.1	Bone fragment	H1	ST5 CC5 type IB	1	0	0	0	1	1	0	1	0	1	0	1	0	0	1	1
83.3	Bursa	H1	ST5 CC5 type IB	0	0	0	0	1	1	0	1	0	1	0	1	0	0	1	1
CACO1	Healthy skin	A1	ST1 CC1 type IA_1_	1	0	0	0	1	1	0	1	0	1	0	1	0	0	1	1
CACO15	Healthy skin	H1	ST42 CC5 type IB	0	0	0	0	1	1	0	0	0	0	0	0	1	0	0	0

ID: identification; ^#^ SLST: single locus sequence typing; ^&^ MLST: multilocus sequence typing; ST: sequence type; CC: clonal complex presence coded as (1); absence coded as (0).

## Data Availability

The datasets presented in this study can be found in online repositories. The names of the repository/repositories and accession number(s) can be found below: https://www.ncbi.nlm.nih.gov/, accessed on 12 September 2023, Genomes accession number JASPKO000000000, JASPKN000000000, JASPKM000000000, JASPKL000000000, JASPKK000000000, JASPKJ000000000, JASPKI000000000, JASPKH000000000, JASPKG000000000, JASPKF000000000, and JASPKE000000000.

## Supplementary Materials

The following supporting information can be downloaded at: https://www.mdpi.com/article/10.3390/microorganisms11102594/s1, Figure S1: Circular genome representation on the CCT (CGView Comparison Tool) map comparing the CDS translations. (A) Comparison of ATCC6919 *C. acnes* to CACO1 (phylotype IA1) genome; (B) Comparison of ATCC11828 to 6.1 (phylotype II) genome; (C) Comparison of Pacn31 to 6.3 (phylotype IA2) genome; (D) Comparison of KPA171202 to 64.1, CACO15, 22.3, 30.3, 34.2, 42.2, 76.1, and 83.3 (phylotype IB) genomes, respectively. Table S1: Susceptibility of antibiotics using MIC tests for 27 isolates of *C. acnes.*; Table S2: List of core and accessory genes with their specific roles found in the *C. acnes* genomes.
