# Supplementary material for: Phenotypic and Genotypic Characterization of Cutibacterium acnes Isolated from Shoulder Surgery Reveals Insights into Genetic Diversity
Source: Microorganisms. 2023 Oct 20;11(10):2594. doi: 10.3390/microorganisms11102594 (PMC10609031; doi:10.3390/microorganisms11102594)
Supplement: Supplementary file 1 [file microorganisms-11-02594-s001.zip › microorganisms-2639542-supplementary.pdf]

SUPPLEMENT MATERIAL

Table S1. Susceptibility antibiotics using MIC test for 27 isolates of *C. acnes*.

Samples identification	TX	VAN	PEN	RIF	LZD	DAP	CLI	MTZ	CIP	AC
**4.3**	<0.016	0.125	0.04	<0.002	0.03	0.047	1	>256	0.5	<0.016
**6.1**	<0.016	0.38	<0.016	0.008	0.18	0.19	0.047	>256	1	<0.016
**6.3**	-	-	-	-	-	-	-	-	-	-
**7.1**	<0.016	0.5	<0.016	<0.002	0.5	0.19	<0.016	>256	0.5	<0.016
**8.2**	0.023	0.5	<0.016	<0.002	0.5	0.23	0.032	>256	1	<0.016
**8.3**	0.032	0.25	<0.016	0.006	0.12	0.19	<0.016	>256	1.5	<0.016
**17.2**	<0.016	0.19	<0.016	<0.002	0.09	0.125	<0.016	>256	1	<0.016
**17.3**	<0.016	0.75	<0.016	<0.002	0.09	0.19	<0.016	>256	1	<0.016
**18.2**	<0.016	0.94	<0.016	0.25	0.12	0.38	<0.016	>256	0.5	<0.016
**22.3**	<0.016	0.25	<0.016	**>32**	0.12	0.5	<0.016	>256	0.75	<0.016
**26.2**	<0.016	0.25	<0.016	<0.002	0.12	0.125	<0.016	>256	1	<0.016
**29.1**	<0.016	0.5	<0.016	0.006	0.09	0.19	<0.016	>256	0.5	<0.016
**29.2**	<0.016	0.25	<0.016	<0.002	0.12	0.19	<0.016	>256	0.75	<0.016
**29.3**	<0.016	0.19	<0.016	<0.002	0.375	0.19	0.032	>256	0.5	<0.016
**30.3**	<0.016	0.125	<0.016	<0.002	0.06	0.064	0.25	>256	**>32**	<0.016
**33.2**	<0.016	0.19	0.003	<0.002	0.25	0.094	1	>256	0.5	<0.016
**34.2**	0.23	0.38	<0.016	0.008	0.12	0.19	<0.016	>256	0.75	0.064
**34.3**	0.094	0.5	0.23	<0.002	0.25	0.19	0.094	>256	1	0.094
**37.1**	<0.016	0.25	<0.016	0.008	0.18	0.19	0.5	>256	0.75	<0.016
**42.1**	0.094	1	<0.016	0.003	0.5	0.25	0.47	>256	0.5	<0.016
**42.2**	0.125	0.5	**0.64**	0.008	0.25	0.25	0.47	>256	0.75	<0.016
**48.3**	<0.016	0.38	<0.016	<0.002	0.03	0.25	<0.016	>256	0.5	<0.016
**49.2**	0.125	0.38	0.023	0.008	0.25	0.25	0.064	>256	1.5	0.032
**55.1**	0.064	0.50	<0.016	0.008	0.5	0.19	0.094	>256	0.5	<0.016
**57.1**	<0.016	0.25	0.003	0.004	0.5	0.19	<0.016	>256	0.25	<0.016
**64.1**	<0.016	0.64	<0.016	<0.002	0.03	0.064	<0.016	>256	1.5	<0.016
**74.1**	<0.016	0.125	<0.016	<0.002	0.03	0.19	0.019	>256	1	<0.016
**76.1**	-	-	-	-	-	-	-	-	-	-
**81.2**	<0.016	0.047	<0.016	<0.002	0.03	0.19	<0.016	>256	0.5	<0.016
**83.3**	-	-	-	-	-	-	-	-	-	-
**BRCAST**										
S	-	≤2	≤0.25	-	-	-	≤4	≤4	-	≤4[4]
I	-	-	0.5	-	-	-	-	-	-	8

R	-	>2	>0.5	-	-	-	>4	>4	-	>8[4]
**CLSI**										
S	≤16	-	≤0.5	-	-	-	≤2	≤8	-	≤4/2
I	32	-	1	-	-	-	4	16	-	8/4
R	≥64	-	≥2	-	-	-	>8	>32	-	≥16/8

MIC= minimal inhibitory concentration, TX = Ceftriaxone, VAN = Vancomycin, PEN = Penicillin, RIF = Rifampicin, LZD = Linezolid, DAP = Daptomycin, CLI = Clindamycin, MTZ = Metronidazole, CIP = Ciprofloxacin, AC = Clavulanic acid/Amoxicillin.

Table S2. List of core and accessory genes with its specific role found in the *C. acnes* genomes.

**Gene**	**Function**	**Classification**	**6.1**	**6.3**	**CA22_3**	**CA30_3**	**CA34_2**	**CA42_2**	**CA64_1**	**CA76_1**	**CA83_3**	**CACO1**	**CACO15**
**MtrA**	Two component system response regulator MtrA	Regulator modulating expression of antibiotic resistance genes											
**LpqB**	Lipoprotein LpqB, modulates activity of MtrAB two-component system	Regulator modulating expression of antibiotic resistance genes											
**MtrB**	Two component system sensor histidine kinase MtrB	Regulator modulating expression of antibiotic resistance genes											
**OxyR**	Hydrogen peroxide-inducible genes activator => OxyR	Regulator modulating expression of antibiotic resistance genes											
**gyrA**	DNA gyrase subunit A (EC 5.99.1.3)	Antibiotic target in susceptible species											
**rpoC**	DNA-directed RNA polymerase beta' subunit (EC 2.7.7.6)	Antibiotic target in susceptible species											
**Ddl**	D-alanine--D-alanine ligase (EC 6.3.2.4)	Antibiotic target in susceptible species											
**kasA**	3-oxoacyl-[acyl-carrier-protein] synthase, KASII (EC 2.3.1.179)	Antibiotic target in susceptible species											
**gyrB**	DNA gyrase subunit B (EC 5.99.1.3)	Antibiotic target in susceptible species											
**folA, Dfr**	Dihydrofolate reductase (EC 1.5.1.3)	Antibiotic target in susceptible species											
**S12p**	SSU ribosomal protein S12p (S23e)	Antibiotic target in susceptible species											
**dxr**	1-deoxy-D-xylulose 5-phosphate reductoisomerase (EC 1.1.1.267)	Antibiotic target in susceptible species											
**EF-G**	Translation elongation factor G	Antibiotic target in susceptible species											
**Iso-tRNA**	Isoleucyl-tRNA synthetase (EC 6.1.1.5)	Antibiotic target in susceptible species											

**inhA, fabI**	Enoyl-[acyl-carrier-protein] reductase [NADH] (EC 1.3.1.9)	Antibiotic target in susceptible species
**rpoB**	DNA-directed RNA polymerase beta subunit (EC 2.7.7.6)	Antibiotic target in susceptible species
**Alr**	Alanine racemase (EC 5.1.1.1)	Antibiotic target in susceptible species
**folP**	Dihydropteroate synthase (EC 2.5.1.15)	Antibiotic target in susceptible species
**rho**	Transcription termination factor Rho	Antibiotic target in susceptible species
**EF-Tu**	Translation elongation factor Tu	Antibiotic target in susceptible species
**S10p**	SSU ribosomal protein S10p (S20e)	Antibiotic target in susceptible species
**rpsL**	SSU ribosomal protein S12p (S23e)	Aminoglycoside resistance gene
**recA**	Adenosine-5'-Diphosphate Monothiophosphate;2'-Deoxyadenosine 5'-Triphosphate	Drug target
**gidB**	16S rRNA (guanine(527)-N(7))-methyltransferase (EC 2.1.1.170)	Gene conferring resistance via absence
**GdpD**	Glycerophosphoryl diester phosphodiesterase (EC 3.1.4.46)	Protein altering cell wall charge conferring antibiotic resistance
**PgsA**	CDP-diacylglycerol--glycerol-3-phosphate 3-phosphatidyltransferase (EC 2.7.8.5)	Protein altering cell wall charge conferring antibiotic resistance
**TadB**	Flp pilus	Flp pilus assembly protein
**ATPase CpaF**	Flp pilus	Flp pilus assembly protein
**CAMP**	cAMP-binding proteins	cAMP-binding proteins
**LuxS**	Autoinducer-2 production protein	Virulence factor

**deoR**	Health-associated strains.	Virulence factor
**ytpA**	Lysophospholipase	Virulence factor
**clpS**	ATP-dependent Clp protease adaptor protein ClpS	Targets specific proteins for destruction in prokaryotes and eukaryotes
**dppB**	ABC transporter	Bacterial growth
gntK	Shikimate kinase	Precursor of aromatic amino acids and secondary metabolites
**HtaA**	Iron acquisition protein	Putative virulence factor genes
**DnaK**	Heat shock proteins	Putative virulence factor genes
**DnaJ**	Heat shock proteins	Putative virulence factor genes
**GroEL**	Heat shock proteins	Putative virulence factor genes
**hyl**	Hyaluronate lyase	Putative virulence factor genes
**DsAs**	Dermatan-sulfate adhesins	Putative virulence factor genes
**PNAG**	poly-β(1-6)-*N*-acetylglucosamine	Biofilm formation
**EF-Tu**	Elongation factor	Biofilm formation
**EF-G**	Elongation factor	Biofilm formation
**rcsB**		Biofilm formation
acsA		Biofilm formation
**YfmO**	Efflux protein	Resistance to MLSB antibiotics
**cse3**	CRISPR-associated endoribonuclease Cse4	CRISPR-Cas systems
**casC**	CRISPR system Cascade subunit CasC	CRISPR-Cas systems
**cas3**	CRISPR-associated nuclease/helicase Cas3	CRISPR-Cas systems
**fucA**	L-fuculose phosphate aldolase	Pathogenesis

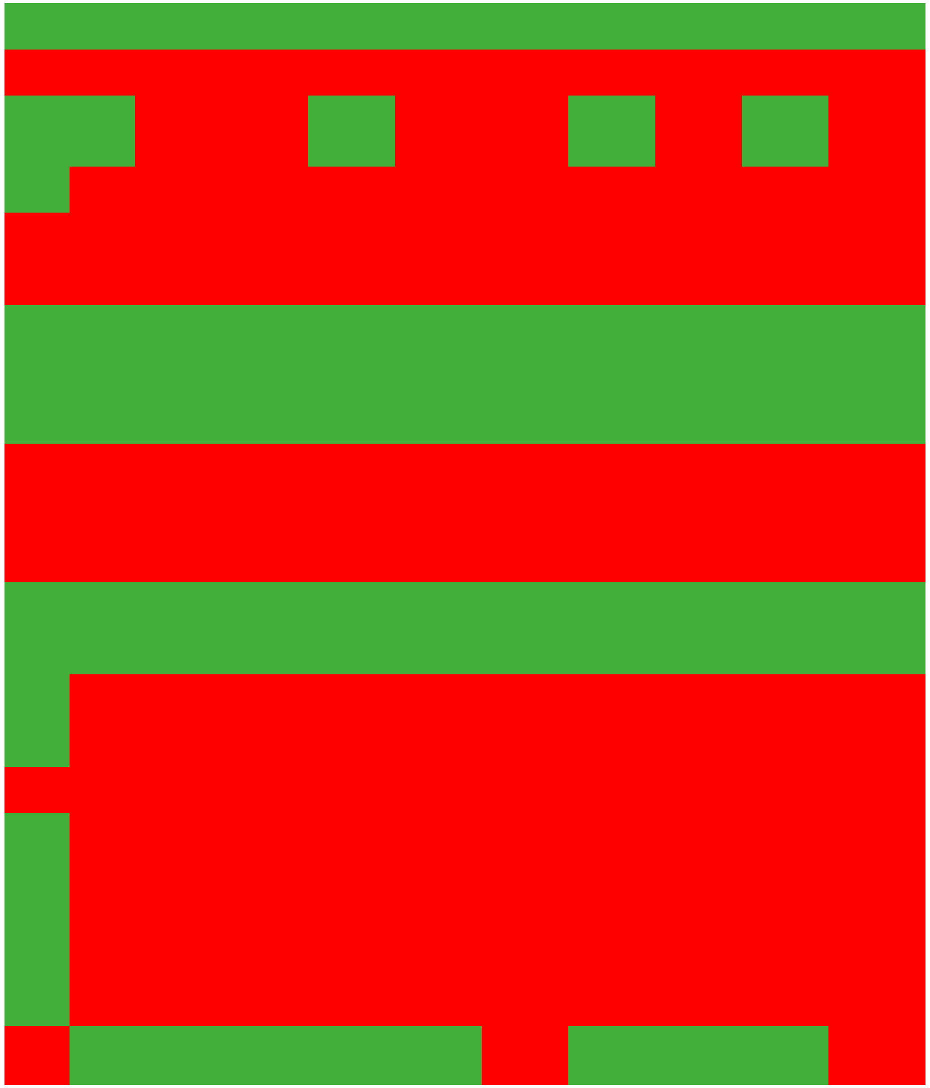

**MprA**	Response regulator MprA	contributes to maintaining a balance among several systems involved in stress resistance and is required for establishment and maintenance of persistent infection in the host
**LiaR**	Transcriptional regulatory protein LiaR	cell envelope stress response
**luxS_2**	S-ribosylhomocysteine lyase	methionine metabolism and quorum sensing
**mprA_1**	Response regulator MprA	maintenance of persistent infection
**qacA**	Antiseptic resistance protein	Resistance

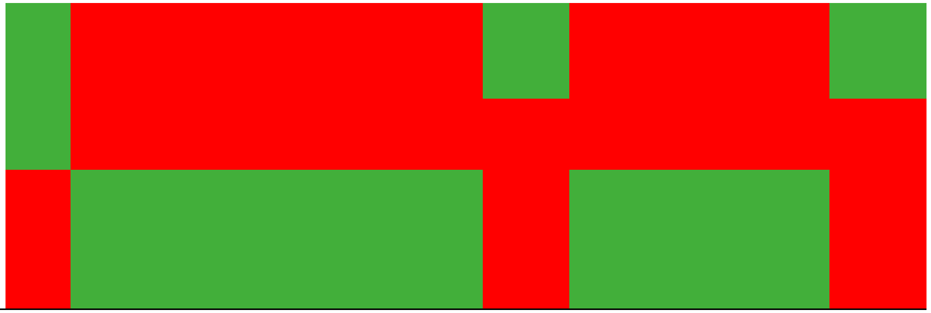

Note: Green color refers to the identification of the gene, while red refers to the absence of the gene.

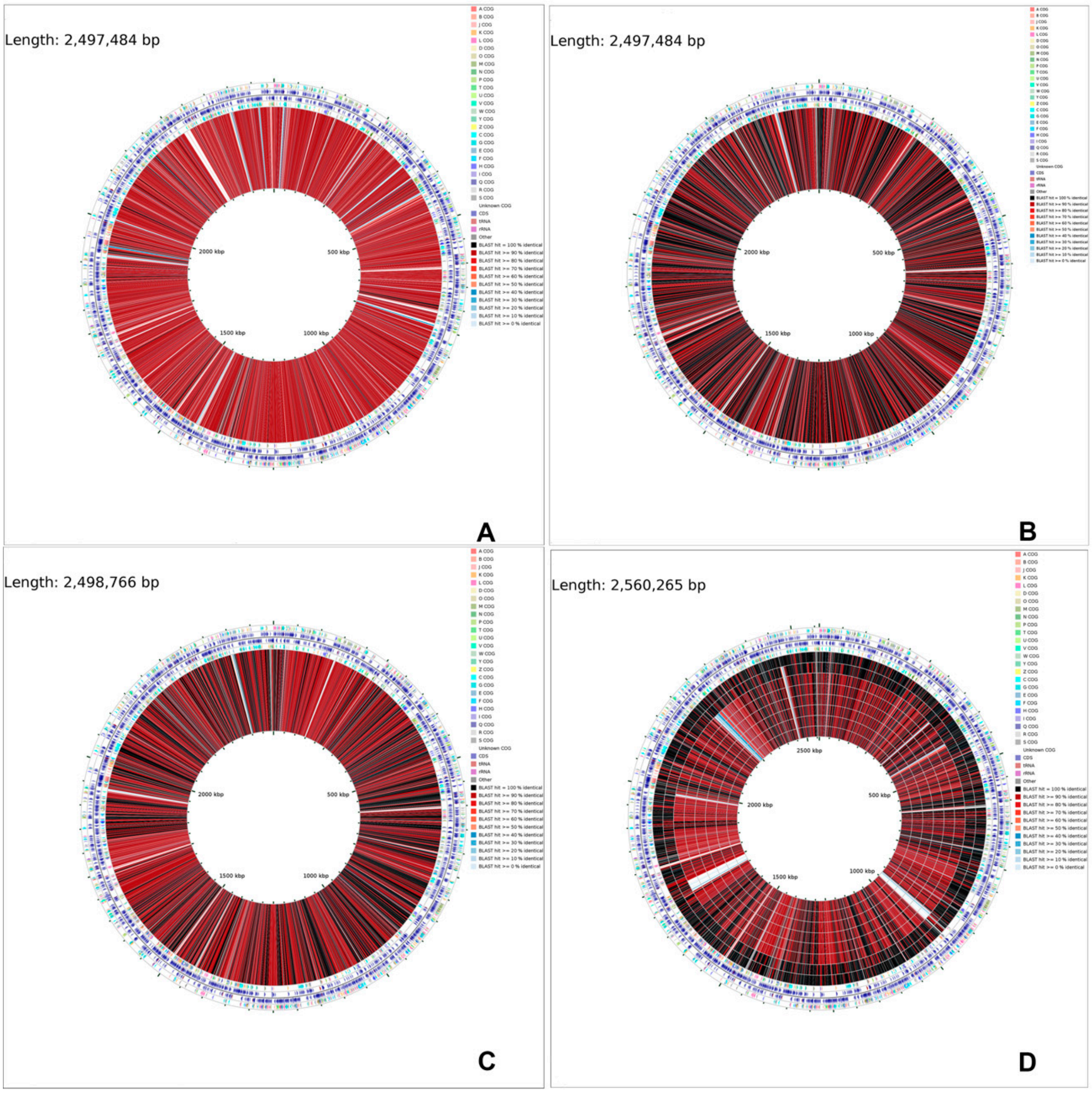

Figure S1. Circular genome representation on the CCT (CGView Comparison Tool) map comparing the CDS translations. (A) Comparison of ATCC6919 *C. acnes* to CACO1 (phylotype IA1) genome; (B) Comparison of ATCC11828 to 6.1 (phylotype II) genome; (C) Comparison of Pacn31 to 6.3 (phylotype IA2) genome; (D) Comparison of KPA171202 to 64.1, CACO15, 22.3, 30.3, 34.2, 42.2, 76.1, 83.3 (phylotype IB) genomes respectively.
